# Combined Single Cell Transcriptome and Surface Epitope Profiling Identifies Potential Biomarkers of Psoriatic Arthritis and Facilitates Diagnosis *via* Machine Learning

**DOI:** 10.3389/fimmu.2022.835760

**Published:** 2022-03-02

**Authors:** Jared Liu, Sugandh Kumar, Julie Hong, Zhi-Ming Huang, Diana Paez, Maria Castillo, Maria Calvo, Hsin-Wen Chang, Daniel D. Cummins, Mimi Chung, Samuel Yeroushalmi, Erin Bartholomew, Marwa Hakimi, Chun Jimmie Ye, Tina Bhutani, Mehrdad Matloubian, Lianne S. Gensler, Wilson Liao

**Affiliations:** ^1^ Department of Dermatology, University of California at San Francisco, San Francisco, CA, United States; ^2^ Division of Rheumatology, Department of Medicine, University of California at San Francisco, San Francisco, CA, United States; ^3^ Institute for Human Genetics, University of California at San Francisco, San Francisco, CA, United States; ^4^ Department of Epidemiology and Biostatistics, University of California at San Francisco, San Francisco, CA, United States; ^5^ Institute of Computational Health Sciences, University of California at San Francisco, San Francisco, CA, United States; ^6^ Parker Institute for Cancer Immunotherapy, San Francisco, CA, United States; ^7^ Chan Zuckerberg Biohub, San Francisco, CA, United States; ^8^ Rosalind Russell/Ephraim P Engleman Rheumatology Research Center, University of California at San Francisco, San Francisco, CA, United States

**Keywords:** psoriatic arthritis, psoriasis, CITE-seq, machine learning, diagnostic test, single cell

## Abstract

Early diagnosis of psoriatic arthritis (PSA) is important for successful therapeutic intervention but currently remains challenging due, in part, to the scarcity of non-invasive biomarkers. In this study, we performed single cell profiling of transcriptome and cell surface protein expression to compare the peripheral blood immunocyte populations of individuals with PSA, individuals with cutaneous psoriasis (PSO) alone, and healthy individuals. We identified genes and proteins differentially expressed between PSA, PSO, and healthy subjects across 30 immune cell types and observed that some cell types, as well as specific phenotypic subsets of cells, differed in abundance between these cohorts. Cell type-specific gene and protein expression differences between PSA, PSO, and healthy groups, along with 200 previously published genetic risk factors for PSA, were further used to perform machine learning classification, with the best models achieving AUROC ≥ 0.87 when either classifying subjects among the three groups or specifically distinguishing PSA from PSO. Our findings thus expand the repertoire of gene, protein, and cellular biomarkers relevant to PSA and demonstrate the utility of machine learning-based diagnostics for this disease.

## Introduction

Psoriatic arthritis (PSA) is an inflammatory rheumatic disease that can affect the peripheral joints, axial joints, and entheses. PSA largely occurs in association with the skin disease psoriasis (PSO), with roughly a third of individuals with PSO developing PSA (1). Early detection of PSA in PSO patients is an important determinant of clinical outcome and patient long-term quality of life (2) but can be challenging due to the heterogeneous presentation of PSA, with only subclinical manifestations at early stages of disease (3).

The ongoing effort to develop better molecular diagnostics for PSA has identified genetic polymorphisms, primarily in major histocompatibility complex and IL-17/IL-23 signaling loci that contribute to PSA risk in PSO patients (4, 5), as well as disease-relevant immune cells within the inflamed synovium of affected joints. These include both adaptive and innate cell types that have a common inflammatory and IL-17-secreting role in pathogenesis and are significantly expanded in the synovium (6). Within peripheral blood, some cell types have also been reported to be perturbed in PSA patients, and while some studies have reported serum biomarkers for distinguishing PSA from PSO (7, 8), a more recent study found similar serum proteomes among PSO patients with and without PSA (9).

In this study, we searched for biomarkers of PSA within the circulating immune cell population by jointly measuring transcriptomic and cell surface protein expression of peripheral blood immune cells at the single cell level. Our data reveal PSA-associated differences in the abundance of phenotypic cell clusters within specific adaptive and innate immune subsets. We further examine disease-associated RNA and protein markers found in this analysis, along with genotype data from PSA-associated polymorphisms, developing a machine-learning-based diagnostic for distinguishing between PSA and PSO.

## Materials And Methods

### Patient Recruitment and Sampling

Patients with PSO were enrolled from the dermatology clinics at the University of California San Francisco (UCSF), with a board-certified dermatologist confirming the clinical diagnosis of plaque psoriasis. Patients with PSA were assessed by a board-certified rheumatologist and diagnosed with PSA according to CASPAR criteria. Patients with psoriasis who reported symptoms of joint pain, but who did not meet CASPAR criteria, were assigned the label of PSX. Healthy controls, who did not have any inflammatory skin disease or autoimmune disease, were enrolled from the San Francisco Bay Area. All subjects gave written, informed consent under IRB approval 10-02830 from the University of California San Francisco. Detailed patient information is provided in **Supplementary Table 1**. Peripheral blood was collected from each subject in Vacutainer ACD tubes. PBMCs were isolated using a standard Ficoll method and stored in liquid nitrogen.

### Sample and Library Preparation

#### Single Cell Libraries

500 µL thawed PBMCs from each subject were added to 10 mL EasySep (StemCell Technologies, Cat. 20144) and centrifuged (300G, 5 min, room temperature). Extracellular nucleic acids were digested by resuspending cell pellets in 1 mL of buffer made from 18 mL EasySep and 21 µL Benzonase Nuclease (MilliporeSigma, Cat. 70664) and incubating (15 min, room temperature). Nuclease-treated cell-suspensions were then filtered through a 40 µm Flowmi Cell Strainer (Bel-Art, Cat. H13680-0040), centrifuged (300G, 5 min, room temperature), and finally resuspended in 100 µL EasySep buffer. Cell counting was performed using a Countess I FL Automated Cell Counter (Thermo Fisher Scientific) on 1:100 dilutions of final cell suspensions stained with 0.4% trypan blue.

#### Cell Surface Staining

Antibody staining of cell surface proteins was performed according to the Totalseq-A protocol (https://www.biolegend.com/en-us/protocols/totalseq-a-antibodies-and-cell-hashing-with-10x-single-cell-3-reagent-kit-v3-3-1-protocol) with modifications as follows.

A pooled suspension containing 2×10^6^ cells from 20 subjects at a time (~100,000 per subject) was centrifuged (300G, 5 min, 4°C) and resuspended in 100 µL Cell Staining Buffer (BioLegend, Cat. 420201) and incubated (10 min, 4°C) with 10 µL Human TruStain FcX™ Fc Blocking Solution (BioLegend, Cat. 422301). Cells suspensions were then stained (30 min, 4°C) with 100 µL TotalSeq antibody cocktail for feature barcoding of cell surface proteins (**Supplementary Table 2**) and divided into two 105 µL aliquots. Each aliquot was washed 3 times by resuspending in 15 mL Cell Staining Buffer and centrifuging (300G, 5 min, 4°C). Aliquots of washed cells were then resuspended in 150 µL 10% FBS in PBS to obtain a concentration of 1×10^6^ cells/mL, recombined, and filtered again with a 40 µm Flowmi Cell Strainer. Cell viability was measured with 10 µL of filtered cells by adding 10 µL 0.4% Trypan Blue and manually counting with a hemocytometer.

Cell density was adjusted to 2,500 cells/µL and run on the Chromium Controller (10X Genomics) using the Single Cell 3’ v3.1 Assay (10X Genomics) with a target of 50,000 cells per reaction.

#### Library Preparation

Gene expression cDNA libraries were prepared according to the manufacturer’s instructions (https://assets.ctfassets.net/an68im79xiti/1eX2FPdpeCgnCJtw4fj9Hx/7cb84edaa9eca04b607f9193162994de/CG000204_ChromiumNextGEMSingleCell3_v3.1_Rev_D.pdf), with 12 cycles of PCR amplification.

Libraries for antibody-derived tags (ADT) from feature barcoding antibodies were prepared by repeating size purification on the supernatant obtained from the prior size purification of gene expression cDNA libraries (Step 2.3.d in the manufacturer’s instructions above), using a 7:8 volumetric ratio of 2.0X SPRIselect reagent (Beckman Coulter, Cat# B23317) to sample. Indexing amplification was performed using Kapa Hifi HotStart ReadyMix (Kapa Biosystems, Cat# KK2601) and TruSeq Small RNA RPI primers (Illumina) with the following thermocycling conditions (1): 98°C, 2 min (2); 15 × (98°C, 20 sec; 60°C, 30 sec; 72°C, 20 sec) (3); 72°C, 5 min. Size purification was then repeated on amplified libraries using a 5:6 volumetric ratio of 1.2X SPRIselect reagent to sample.

Libraries were quantified using a Bioanalyzer 2100 (Agilent) and sequenced on a Novaseq 6000 (Illumina).

#### Genotyping

DNA for genotyping was extracted from whole blood using the DNeasy blood and tissue kit (Qiagen, Cat. 69504). Extracted DNA was genotyped on the Affymetrix UK Biobank Axiom Array (ThermoFisher) using a GeneTitan Multi-Channel Instrument (Applied Biosystems).

### Genotype Data Processing

SNPs were called using Analysis Power Tools 2.10.2.2 (Affymetrix, https://www.affymetrix.com/support/developer/powertools/changelog/index.html). The resulting genotype.vcfs were scanned with ‘snpflip’ (https://github.com/biocore-ntnu/snpflip) using the GRCh37 build of the human genome reference sequence maintained by the University of California, Santa Cruz (http://hgdownload.cse.ucsc.edu/goldenPath/hg19/bigZips/hg19.fa.gz) to identify reversed and ambiguous-stranded SNPs, which were flipped and removed (respectively) using Plink 1.90 (http://pngu.mgh.harvard.edu/purcell/plink/) (10), and the remaining sites were sorted using Plink 2.00a3LM (www.cog-genomics.org/plink/2.0/) (11). This SNP data was then augmented with additional sites imputed by the Michigan Imputation Server (https://imputationserver.sph.umich.edu) (1000G Phase 3 v5 GRCh37 reference panel, rsqFilter off, Eagle v2.4 phasing, EUR population). SNP positions were translated to GRCh38 coordinates using the ‘LiftoverVcf’ command of Picard 2.23.3 (http://broadinstitute.github.io/picard/). Finally, Vcftools 0.1.13 (12) was used to exclude non-exonic SNPs and SNPs with minor allele frequency < 0.05.

### Single Cell Data Processing

Raw RNA and ADT fastqs for each Chromium library were respectively aligned to the GRCh38 human genome reference and the antibody-tag reference (**Supplementary Table 2**) using Cell Ranger 3.1.0 (10X Genomics) with default settings to obtain RNA and matched ADT (if available) count matrices for all barcodes representing non-empty droplets.

#### Cell Demultiplexing, Doublet Removal, and Annotation

Within each RNA count matrix, the subject of origin for all droplet barcodes was determined by using ‘demuxlet’ (13), as implemented in the ‘popscle’ suite (https://github.com/statgen/popscle), with imputation-augmented exonic SNP genotypes described above, and doublets detected between different individuals were excluded. The count matrices for each Chromium library were then loaded into R for analysis using the ‘Seurat’ 4.0.3 (14) R package, and the ‘DoubletDecon’ 1.1.6 R package (15) was used to further remove doublets formed by different cells within the same individual.

#### QC and Cell Annotation

Cell type annotation was performed by integrative mapping of annotations from a previously published dataset of 161,764 healthy PBMCs (14) onto our dataset. Specifically, we used the ‘TransferData’ Seurat function according to the Seurat protocol (https://satijalab.org/seurat/reference/transferdata) to transfer annotations for 30 distinct cell types from the ‘predicted.celltype.l2’ metadata variable.

We performed filtering of cells based on both RNA and ADT data by retaining cells with total RNA unique molecular identifiers (UMIs) between 500 and 10,000, total RNA features ≥ 200, percent mitochondrial and ribosomal protein reads in RNA ≤ 15% and 60% (respectively), total ADT features ≤ 260, and percent ADT reads mapping to 9 isotype control antibodies < 2%. In the RNA matrices of the resulting data, we further removed features (genes) with no detectable UMIs across the cells of all matrices. These matrices were finally merged into a combined matrix of RNA data for all cells. In the ADT matrices, we further removed features corresponding to the 9 isotype controls and 15 features observed to have expression inconsistent with annotated cell types (**Supplementary Table 2**). Lastly, we observed that a single healthy subject was represented by only 4 cells after filtering. These cells were excluded from later analysis.

#### ADT Imputation and UMAP Generation

ADT expression was estimated for cells with measured RNA but not ADT according to the Seurat reference mapping protocol (https://satijalab.org/seurat/articles/multimodal_reference_mapping.html), and unless otherwise noted, all function names described here belong to the Seurat package. Briefly, the integrated dataset above was split into the subset of cells with ADT measurements (reference subset) and the subset of cells without ADT measurements (query subset). RNA expression normalization and scaling were performed using ‘SCTransform’ on both subsets, adjusting for the number of features and total counts in each cell *via* the ‘vars.to.regress’ parameter. ADT expression normalization for the reference subset was performed using the centered log ratio (CLR), followed by mean centering and scaling. For the reference subset, PCA was then run for both the SCTransformed RNA (SCT) expression and the ADT expression, and a weighted nearest-neighbor network for the reference subset was calculated from the first 30 and 18 PCs for SCT and ADT, respectively, using the ‘FindMultiModalNeighbors’ function. Next, SCT from the reference subset was transformed again using supervised PCA (via the ‘RunSPCA’ function) to identify the principal components that best capture the combined RNA and ADT expression variation represented by the weighted nearest-neighbor network.

The first 50 components of this transformation were then used to identify anchors between the reference subset and the SCT of the query subset using the ‘FindTransferAnchors’ function. Finally, imputed ADT (ADTimp) data for the query subset was calculated using the ‘TransferData’ function. A weighted nearest-neighbor network was calculated using both SCT and ADTimp according to the Seurat protocol (https://satijalab.org/seurat/articles/weighted_nearest_neighbor_analysis.html).

#### Intra-Cell Type Differential Feature Analysis

To identify differentially expressed genes (DEGs) and proteins (DEPs), the Seurat object containing ADT and RNA expression from the QC’d dataset (see section QC and Annotation above) was subsetted by annotated cell type using ‘SplitObject’. For each resulting Seurat object containing cells of a particular type, we performed normalization on RNA expression using SCTransform, again adjusting for processing batch (‘Run’ metadata variable) within each cell type (using the ‘vars.to.regress’ parameter of SCTransform). Differential gene expression between disease statuses as well as between clusters (see section ‘*Intra-cell type clustering’*) was then calculated on SCTransform-normalized counts using the negative binomial test (test.use = “negbinom” in Seurat). Genes with both Bonferroni-corrected p-value < 0.05 and absolute log fold change > 0.25 were considered significant. Differential protein analysis was performed similarly, except with the Wilcoxon test (test.use = “wilcox” in Seurat) on CLR-normalized, mean-centered and scaled ADT data (within the ‘scale.data’ slot of the Seurat object) only for cells with measured (i.e. non-imputed) ADT data.

#### Cell Type Proportion Comparison

To detect statistical differences in the frequencies of each annotated cell type between cohorts, we calculated, for each cell type, the proportion of cells of that cell type in each subject out of the total number of cells in the subject, and the Kruskall-Wallis test (‘kruskal.test’ in R) was used to determine whether significant cell proportion differences existed between any cohort of subjects. For cell types with FDR-adjusted Kruskall-Wallis p-values < 0.05, we then performed Wilcoxon tests (‘wilcox.test’ in R) to identify significant (unadjusted p-value < 0.05) differences in cell proportions between cohorts. The same method was used to test for differences in the proportions of subclusters within cell types.

#### Intra-Cell Type Clustering

To identify phenotypic clusters within cell types, the RNA expression data for a cell type was first corrected for batch effects by first subsetting the raw count matrix by the cells within each sequencing batch. SCTransform was run individually for each count matrix, and the resulting SCT expression matrices were reintegrated into a single matrix (see section ‘*Data integration’*). PCA was performed on the integrated SCT matrix, and the first 30 PCs were used to construct a shared nearest-neighbor network using the ‘FindNeighbors’ function. The network was then used to identify clusters with the ‘FindClusters’ function, using a resolution of 0.6. UMAPs were also generated from the first 30 PCs using the ‘RunUMAP’ function.

#### Data Integration

Integration of SCT expression data from two or more single-cell datasets was performed according to the Seurat data integration protocol (https://satijalab.org/seurat/articles/integration_introduction.html#performing-integration-on-datasets-normalized-with-sctransform-1). Briefly, ‘SelectIntegrationFeatures’ was used to select a common set of 3,000 genes most consistently variable among the individual SCT matrices, and ‘PrepSCTIntegration’ was then used to prepare reduced SCT expression matrices for just these genes. PCA was calculated for each reduced SCT matrix using ‘RunPCA’, and the first 50 principal components of this transformation were used to identify transcriptionally similar cells between each pair of reduced SCT matrices using ‘FindIntegrationAnchors’, with ‘reduction’ set to ‘rpca’. Finally, an integrated SCT matrix was calculated using ‘IntegrateData’.

### Machine Learning Model Development

Input data for classifying each subject in PSA, PSO, healthy, and PSX cohorts was prepared by calculating the mean of the normalized, centered, and scaled expression of each feature in the set of cell type-specific differentially-expressed genes and proteins (found between PSA and healthy, PSO and healthy, and PSA and PSO groups; see section *‘Intra-cell type differential feature analysis*’) for all cells of the corresponding cell type in a given subject. The feature expression data for healthy, PSA, and PSO subjects (N=81) were then divided into a training set, n=58 (healthy=21, PSA=20, PSO=17) and a test set, n=23 (healthy=8, PSA=8, and PSO=7) to achieve a training:test ratio of 70:30.

We first performed ensemble-based feature selection using the EFS-MI method (16) where subsets of the starting feature set predicted to be informative by four different ML algorithms (Feed Forward and Backward selection, Recursive RF, SVMRadial, and NNET) were combined and sorted by prediction potential classification rank. We selected the top twenty features to train eleven ML algorithms such as linear, non-linear, and ensemble provided by the ‘caret’ R package, assessing classification performance using accuracy and kappa. To avoid overfitting and reduce the noise of random fitting models, we employed 10-fold cross-validation with 1,000 iterations. We selected Random Forest (RF), Support Vector Machine Radial Kernel (SVMRadial), and Neural Network (NNET) algorithms for test set validation, based on the suitability of our data set, popularity, and reliability. For the RF model, tuning parameters were optimized with bootstrap = TRUE, which resembles random sampling during model building. The maximum number of tree splits in each step was a max_depth = (50, 80, 100, 150, 300), maximum features were selected as auto (max_features = ‘auto’), and for error minimization through impurity value (min_impurity_decrease = c(0.0, 0.02, 0.1, 0.5). Next, a minimum tree split as a leaf in each step (min_samples_leaf = (1 to 10), maximum generation of trees (n_estimator = 20), and other parameters as a default. The best fit optimized parameters were considered the final model for further evaluation. For Support Vector Machine (SVM), we tuned two major parameters: 1) cost function, which ensures the decision boundary for data classes, and 2) the sigma value, which defines how much influence a single training set has on the model, with lower sigma and cost resulting in better prediction accuracy. For neural network algorithms, we used hidden layers (size = 1,2,4,6,10,15) and learning rate (decay = 0, 0.05, 0.1, 1, 2) as tuning parameters (17). The prediction statistics and accuracy of RF, SVMRadial and NNET were examined through several statistics such as Area Under the Receiver Operating Characteristic (AUROC), balanced accuracy (kappa), sensitivity, and specificity, which are compiled in **Supplementary Table 3**.

The genotypes of each subject at each of the 200 PSA-associated SNPs identified by Patrick et al. (18) were compiled from imputed subject genotyping data (see section *‘Genotype data processing*’). We coded genotypes homozygous for the non-risk allele as zero, heterozygous as one, and homozygous for the risk allele as two. As above, eleven ML algorithms were trained on this data and evaluated based on classification accuracy and kappa, and the performance of three models (RF, SVMRadial, and NNET) were examined through test set data and optimized using the same tuning parameters. The ML algorithms were run with set.seed=862 for reproducibility of models.

## Results

### Cell Types Enriched and Depleted Among PSA, PSO, and Healthy PBMCs

We characterized the differences in cellular composition as well as transcriptional and cell surface protein expression between 28 PSA, 24 PSO, and 29 healthy subjects, along with 14 psoriasis patients with unclear PSA diagnosis (PSX) by performing single cell RNA-seq on PBMCs, obtaining transcriptomes of 392 – 7003 (median of 2,392) cells per subject (total 246,762 cells). For a subset of these cells (133,665, 54%), we additionally performed antibody-derived tag labeling of 258 cell surface proteins (**Supplementary Table 2**).

**Figure 1:**
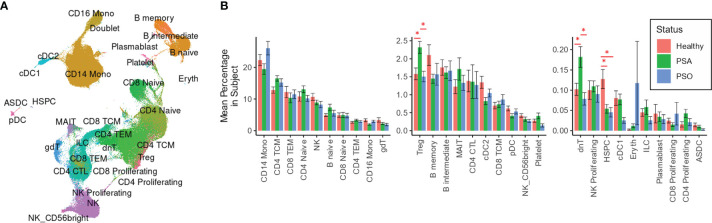
Cell types and subsets among PSA, PSO, and healthy individuals. **(A)** UMAP of SCTransform-normalized RNA expression integrated with ADT expression, colored by cell subset. **(B)** Mean percentages of each cell type within the total PBMCs of each subject. Error bars indicate standard error of the mean; * indicates both Wilcoxon and FDR-adjusted Kruskall-Wallis p-values < 0.05.

We performed integrative mapping of transcriptomic data from our cell population to categorize all cells into 30 phenotypic subsets defined in a previously described multimodal reference dataset of healthy PBMCs (**Figure 1A**) (14). All 30 cell types were comparably represented among PSA, PSO, PSX and healthy subjects (**Figure 1B**), with the exception of Tregs and dnT cells, which were relatively increased in PSA patients compared to both PSO and healthy subjects (p < 0.03, **Figure 1B**), and hematopoietic stem precursor cells (HSPCs), which were relatively increased in healthy subjects (p < 0.007).

### Gene and Protein Biomarkers of PSA Include Activational and Metabolic Transcriptomic Differences That Distinguish PSA From PSO

**Figure 2:**
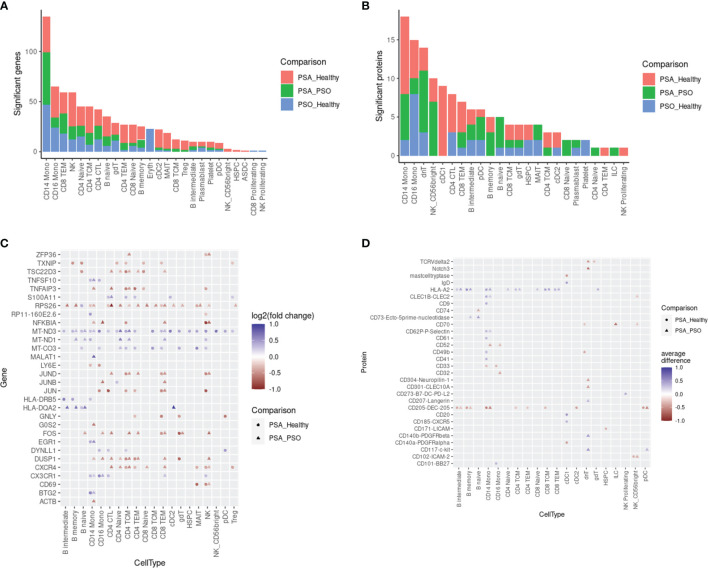
Differentially expressed features between PSA, PSO, and healthy subjects within cell types. Counts of differentially expressed **(A)** genes and **(B)** cell surface proteins are shown for each comparison within each cell type. Top 30 differentially expressed **(C)** genes and **(D)** cell surface proteins in each cell type are ranked by highest absolute log2 fold change (for genes) or absolute mean difference (for proteins) between PSA cells vs. PSO (circles) or healthy (triangles) cells.

We next surveyed the phenotypic differences between PSA, PSO, and healthy cells of each cell type by calculating differentially expressed genes (DEGs) and proteins (DEPs). Within each cell subset, we found 1 – 135 DEGs (median 23) and 1 – 18 DEPs (median 4) with significant differences between PSA and PSO, PSA and healthy, or PSO and healthy cells (**Figures 2A, B**), with the most differentially expressed features detected in CD14 monocytes, the most abundant cell type in our dataset.

The DEGs and DEPs represented both broad as well as cell type-specific disease-associated expression differences. Among 30 DEGs with the highest absolute fold change (**Figure 2C**), we observed a general upregulation of mitochondrial genes (*MT-CO3, MT-ND1*, *MT-ND3*) paired with a downregulation of ribosomal protein gene *RPS26* across most cell types in PSA patients relative to PSO patients or healthy subjects. Among PSA T and NK cells, we also observed a downregulation of AP-1 transcription factors (*JUN, JUNB, JUND, FOS*) and regulators of activation (*TNFAIP3, DUSP1*) along with the upregulation of *S100A11*, a calcium-binding protein associated with rheumatoid arthritis (19). Lastly, we observed PSA-associated differences in chemokine receptor expression, specifically a downregulation of *CXCR4* in T and NK cells and an upregulation of *CX3CR1* in monocytes, NK cells, and specific T cell subsets. Disease-associated differences in cell surface protein expression, in contrast, were more sparsely observed within specific cell types (**Figure 2D**). Among the top 30 DEPs, HLA-A2 was broadly upregulated among B and T cell subsets as well as CD14 monocytes in PSA patients, while CD205 was broadly downregulated in many of the same cell types along with cDC2 and pDC subsets.

### Phenotypic Subsets of Specific Cell Types Enriched and Depleted in PSA

**Figure 3:**
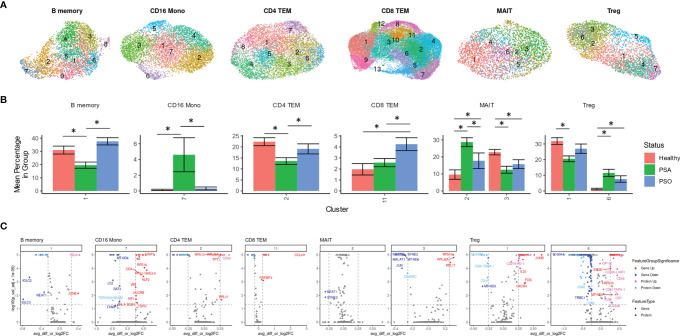
Immune cell subsets differentially abundant in psoriatic and healthy individuals. **(A)** UMAP of *de novo* clusters identified within select cell types containing clusters with significant abundance differences. **(B)** Average percentage of cells from each PSA, PSO, or healthy subject in a given cluster out of total cells from that subject in the given cell type. **(C)** Volcano plots of genes and cell surface proteins upregulated and downregulated in each cluster relative to other cells of the same cell type. * indicates Wilcoxon p-value < 0.05.

Besides differences in cellular composition and gene or protein expression, we also searched for additional disease signatures of PSA among the phenotypic subsets of each cell type. By performing integrative, transcriptome-based *de novo* clustering of each cell type with at least 1,000 cells, we identified phenotypic clusters within six cell types that were enriched in different disease conditions (**Figure 3A**).

Some of these phenotypic subsets were uniquely associated with PSA. Within CD16 monocytes, a small cluster (cluster 7) was more abundant among PSA subjects (**Figure 3B**). Compared to other CD16 monocytes, the 108 cells of this cluster showed generally lower expression of several mitochondrial genes (**Figure 3C** and **Supplementary Table 5**) and higher expression of S100 genes (*S100A4, S100A6, S100A10, S100A11*), as well as genes involved in antigen presentation (*HLA-DRB5*, *HLA-DQB1, FCER1G*) and regulation of innate activation [*DUSP1* (20)]. We also observed a cluster of PSA-abundant MAIT cells (cluster 2), however, these cells may potentially represent a clustering artifact, as no significantly over- or under-expressed genes were found to distinguish this cluster from other cells. Analysis of differentially expressed proteins in these two clusters yielded a single protein, Tetraspanin 33, which was under-expressed in CD16 monocyte cluster 7.

On the other hand, we also found clusters uniquely reduced in PSA within B memory (cluster 1) and a CD4 TEM (cluster 2) cells (**Figure 3B**). The B memory cluster was characterized by a small number of gene expression differences including reduced expression of *IGLC2* and *IGLC3* that was consistent with downregulated cell surface expression of immunoglobin light chain protein (**Figure 3C** and **Supplementary Table 5**). Additionally, we observed increased expression of *JUNB*, a negative regulator of growth and proliferation (21) and downregulated expression of transferrin receptor [CD71, a marker of activated or proliferating B cells (22)] and several other receptors found to promote apoptosis and proliferation [CD95 (23), CD164 (24)] or response to chemokines (CD99 (25)]. The CD4 TEM cluster showed a downregulation of *DUSP2*, a negative regulator of Th17 differentiation (26), as well as Jun/Fos genes (*JUN, JUNB, FOS, FOSB*) and, unexpectedly, several genes associated with cytotoxic function (*GZMA, GZMK, NKG7, SRGN*) (**Supplementary Table 5**). Differential protein analysis revealed an upregulation of gut-homing integrin β7 and receptors that promote cell proliferation [CD55 (27)] and maintain T cell survival [CD127 (28)].

Other clusters were associated specifically with PSO or healthy subjects. Cells within a single CD8 TEM cluster enriched among PSO subjects (cluster 11, **Figure 3B**) showed a strong upregulation of *CCL4*, a CD8^+^ T cell recruiting (29) chemokine associated with psoriasis (30), along with other inflammatory cytokines and chemokines (*TNF, IFNG, CCL3, CCL4L2*) (**Figure 3C** and **Supplementary Table 5**). Differential expression analysis of cell surface proteins on this cluster revealed an upregulation of GPR56, a marker of cytotoxic cells (31) as well as reduced expression of chemokine receptor CXCR3. We also found a MAIT cluster (cluster 3) enriched among healthy subjects, though, similar to MAIT cluster 2 above, these cells are distinguished by relatively few markers that included ribosomal proteins and long non-coding RNAs *NEAT1* and *MALAT1* (**Figure 3C** and **Supplementary Table 5**).

Lastly, clustering analysis among Tregs revealed an imbalance of resting and activated Tregs between healthy and psoriatic (PSO and PSA) subjects. Differentially expressed genes in a Treg cluster enriched in PSA (cluster 6, **Figure 3B**) consisted of an upregulation of 52 genes that mostly encoded ribosomal proteins and a downregulation of 115 genes, including some involved in class I and class II antigen presentation (*HLA-A, HLA-B, HLA-C, HLA-E, HLA-DPA1, HLA-DPB1, HLA-DRA, HLA-DRB1*) and *CD52* (**Supplementary Table 5**), which encodes a costimulatory receptor found to promote Treg suppression of CD4 and CD8 T cells (32). Differential expression of cell surface proteins also revealed a lower expression of memory marker CD45RO, which, combined with higher expression of CD45RA and *CCR7* in this cluster (**Figure 3C** and **Supplementary Table 5**), suggests a naïve, or antigen-inexperienced state. We additionally observed an upregulation of GP130 (**Figure 3B**), a subunit of multiple cytokine receptors such as IL-6R that has been found to define a Treg subset with reduced suppression function (33). These protein expression differences were reversed in the relatively healthy-enriched cluster 1, in which GP130 and CD45RA were reduced in expression while CD45RO, along with costimulatory markers such as TIGIT and PD-1, were increased in expression. DEGs from this cluster, including an upregulation of *DUSP1*, *CXCR4* and Jun and Fos family genes (**Figure 3C** and **Supplementary Table 5**) further suggested an activated, functionally suppressive phenotype, and *FOXP3* expression was higher (though not significantly) in this cluster than cluster 6 (**Supplementary Table 5**).

### Machine Learning Classifiers Distinguish Between PSA, PSO, and Healthy Subjects Using Cell Type-Specific DEGs and DEPs

We evaluated the diagnostic potential of the PSA-associated DEGs and DEPs by using them to perform ML classification of subjects in our study cohort. Based on the cell-type specific mean expression of 257 DEGs and 258 DEPs (**Supplementary Table 4**) averaged within each subject’s cells in the corresponding cell types, we performed ensemble feature selection (16) using four ML algorithms to identify the top twenty DEGs and DEPs based on their classification rate (Importance).

**Figure 4:**
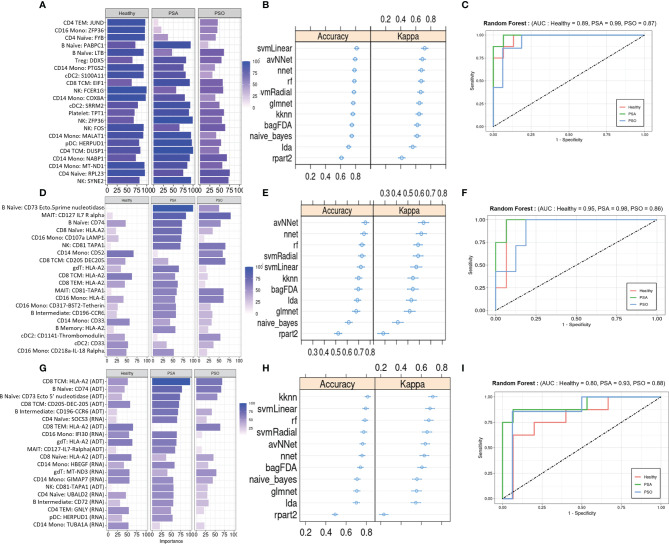
Machine learning classification of healthy, PSA and PSO subjects. **(A)** Classification rate (Importance) of top 20 DEGs, along with corresponding **(B)** accuracy and kappa of eleven different ML classifiers trained on these features. **(C)** ROC curve of RF model for healthy, PSA, and PSO classification. Analogous plots shown for **(D–F)** DEPs and **(G–I)** DEGs combined with DEPs. Error bars indicate 95% confidence interval.

The top twenty DEGs span a variety of immune cell types, and many encode proteins involved in metabolism, translation, and transcriptional regulation (**Figure 4A**). 10-fold cross validation of eleven ML algorithms trained on these features classified PSA, PSO, and healthy subjects with average accuracies of 0.65 – 0.89 (**Figure 4B**) across 1,000 iterations. Kappa, a measure of the agreement between observed and expected accuracy ranged from 0.41- 0.72 across the eleven algorithms. Further evaluation of the sensitivity and specificity of the RF model demonstrated an AUROC of 0.89, 0.99, and 0.87 for healthy, PSA, and PSO subjects (**Figure 4C**) with similar results for SVMRadial and NNET models (**Supplementary Figure 4**).

Similar to the top twenty DEGs, the top twenty DEPs spanned several cell types but showed generally lower classification Importance (**Figure 4D**). Accordingly, the average accuracy of the eleven ML algorithms on the top twenty DEPs (0.58 - 0.86, **Figure 4E**) and kappa (0.21 - 0.65) were relatively lower than for DEGs, with similarly reduced AUROC for RF (Healthy = 0.80, PSA = 0.93, PSO = 0.88, **Figure 4F**), SVMRadial, and NNET (**Supplementary Figure 4**).

To test whether classifier performance could be improved by considering both gene and cell surface protein expression together, we also performed ensemble feature selection on the combined expression data of DEPs and DEGs from the above analyses. The resulting set of twenty features consisted of 10 DEGs and 10 DEPs spanning several cell types, with similar classification Importance measures as the set of DEPs only (**Figure 4G**). Classification accuracies of the eleven ML algorithms based on this feature set were more comparable to those of DEGs alone (accuracy 0.68 - 0.89, kappa 0.54 – 0.76, **Figure 4H**), except for rpart which performed worse on this feature set than on DEGs and DEPs separately (average accuracy 0.52, kappa 0.26). AUROC for RF was relatively lower than DEP- and DEG-only models for healthy (0.80) and PSA (0.93) groups but similar to those models for PSO (0.88) subjects (**Figure 4I**), with comparable results for SVMRadial and NNET models (**Supplementary Figure 4**).

We performed further validation of the RF model by using it to classify a cohort of 14 subjects (PSX) presenting with cutaneous psoriasis and joint pain that did not confidently meet current PSA diagnosis criteria. RF classification based on DEGs, DEPs, or both consistently categorized 10 of these patients as PSA and one patient as healthy (**Supplementary Figure 5** and **Supplementary Table 6**), with the remaining three subjects discordantly classified as healthy or PSO.

### ML Classifiers Detect PSA in Psoriatic Individuals Using DEGs, DEPs, or Genetic Risk Factors

**Figure 5:**
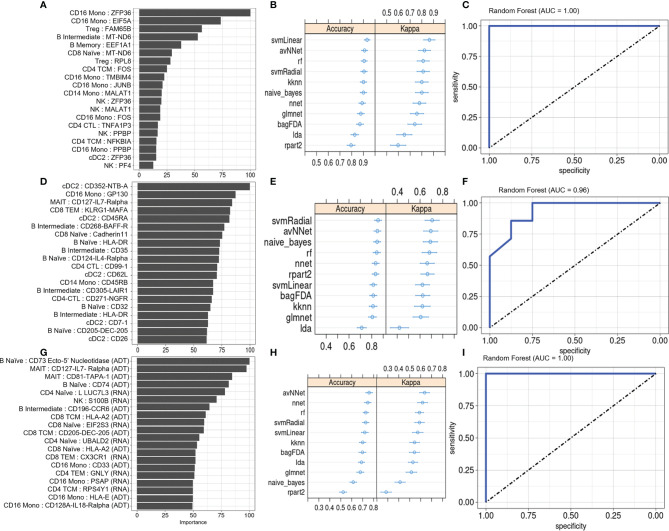
Machine learning classification of PSA vs. PSO subjects. **(A)** Classification rate (Importance) of top 20 DEGs, along with corresponding **(B)** accuracy and kappa of eleven different ML classifiers trained on these features. **(C)** ROC curve of RF model. Analogous plots shown for **(D–F)** DEPs and **(G–I)** DEGs combined with DEPs. Error bars indicate 95% confidence interval.

We also evaluated the diagnostic potential of DEGs and DEPs, separately or in combination, for detecting PSA among individuals presenting with cutaneous psoriasis by performing a two-way classification of PSA and PSO groups. As before, the top twenty DEGs and DEPs were associated with several immune cell types, with the DEG set including many genes with roles in metabolism and in the regulation of activation and inflammation (**Figure 5A**). Among PSA and PSO subjects, we noted higher Importance measures among the top twenty DEPs compared with the top twenty DEGs (**Figures 5A, D**), however performance metrics of the eleven ML models were generally higher in DEGs (accuracy 0.81 – 0.94, kappa 0.41 – 0.83) than DEPs (accuracy 0.73 – 0.92, kappa 0.42 – 0.72, **Figures 5B, E**). In addition, RF, SVMRadial, and NNET all achieved perfect classification of PSA and PSO subjects using DEGs (AUROC of 1, **Figure 5C** and **Supplementary Figures 6A, B**) compared to the slightly lower classification performance for DEPs (**Figure 5F** and **Supplementary Figures 6C, D**). Feature selection on combined DEPs and DEGs yielded a top twenty feature set with Importance measures that were intermediate between the sets of DEPs and DEGs alone (**Figure 5G**), and while ML classifier performance was lower for the combined feature set (accuracy 0.52 – 0.81, kappa 0.26 – 0.67, **Figure 5H**), AUROC for the RF and SVMRadial models (1.00 and 0.96, respectively, **Figure 5I** and **Supplementary Figure 6E**) was comparable to those of DEG- and DEP-only feature sets, with NNET underperforming substantially (AUROC 0.7, **Supplementary Figure 6F**).

**Figure 6:**
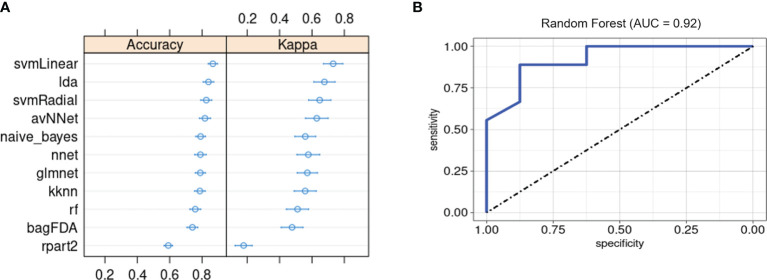
Machine learning classification of PSA vs. PSO subjects based on 200 PSA-associated genetic risk loci. **(A)** Accuracy and kappa of eleven ML models. **(B)** ROC curve for RF model. Error bars indicate 95% confidence interval. Error bars indicate 95% confidence interval.

Lastly, we evaluated whether our ML framework for detecting PSA in a background of cutaneous psoriasis could also be applied to genetic biomarkers of PSA risk. ML classifiers trained on patient genotypes at 200 SNP sites previously found to be associated with PSA (18) achieved average classification accuracies between 0.6 and 0.87 and kappa between 0.51 and 0.73 (**Figure 6A**). AUROC of RF, SVM-Radial, and NNET was 0.92 (**Figure 6B**), 0.88, and 0.81, similar to metrics reported in the previous study (18).

## Discussion

Our study sheds light on the phenotypic differences between the circulating immune cells of PSA and PSO patients at multiple levels of resolution. At the cellular level, we observed a higher abundance of Tregs and dnT cells in PSA patients and a higher abundance of HSPCs in healthy subjects. While, to our knowledge, the role of dnTs and HSPCs in PSA has not been extensively investigated, dnT cells have been reported to infiltrate psoriatic skin as well as participate in IL-23/IL-17 signaling in mouse models of psoriasis (34) and spondyloarthritis (35), and the proliferation and differentiation of HSPCs is currently known to respond to systemic interferon and TNF signaling (36). While we observed increased peripheral Tregs in PSA patients, whether this subset is generally increased or decreased in PSA is still unclear in light of conflicting results found in other studies (37, 38).

Within each cell type, our *de novo* clustering analyses identified disease-associated subsets and potential biological processes affecting them. First, the skewing of peripheral Tregs toward more naïve, resting cells and fewer activated effector cells in PSA and PSO parallels what has been observed in systemic lupus erythematosus (39) and could reflect either a migration of effector Tregs from circulation into sites of inflammation or a general expansion of the naïve Treg pool. Second, our study also identified a cluster of CD8 TEM cells specific to PSO but not PSA. The strong upregulation of *CCL4* coupled with the downregulation of CXCR3 in this cluster raise the possibility that differences in chemokine-mediated immune cell homing (e.g. to skin compared with synovium) could emerge as a key characteristic for predicting PSA progression or risk in PSO patients, especially in light of evidence suggesting that CXCR3 may be involved in T cell recruitment in PSA, based on higher protein expression of its ligands, CXCL9 and CXCL10, in synovial compared with peripheral compartments (40, 41), whereas no such difference was found for MIP1β, the chemokine encoded by *CCL4* (41). Besides the overall PSA-associated downregulation of *CXCR4* and upregulation of *CX3CR1* observed in our data, other studies have identified PSO- and PSA-associated T cell subsets expressing CCR5 (42), CCR4 (43), and CCR10 (37), and the questions of whether and how signaling through these chemokine receptors mediates trafficking of pathological T cells between the skin, blood, and joint remain active areas of investigation. Lastly, the enrichment of a CD16 monocyte subset that we observed in PSA subjects is consistent with previous findings of increased circulating CD16+ monocyte population in PSA subjects that can give rise to osteoclasts (44). Other studies in mice have found that subsets of other myeloid cell types, such as neutrophils, may also contribute to psoriatic disease through T-cell independent responses to IL-17A signaling (45, 46).

At the molecular level, we found disease-associated protein and gene expression signatures within diverse innate and adaptive immune cell types, consistent with the current understanding that multiple cell types contribute to inflammation in PSA (6). While these contributions have mainly been investigated in the context of IL-17 and IL-23 signaling, our data sheds light on other characteristics that distinguish circulating immunocytes in PSA patients, such as generally increased mitochondrial gene expression and decreased expression of cell activational regulators. Although disease conditions may generally alter protein and gene expression divergently among different cell types, we note that, in our data, gene and protein expression in different cell types are largely perturbed in the same direction by PSA (i.e. a feature upregulated in PSA cells of one type is generally upregulated in PSA cells of other types).

Importantly, our study demonstrates the utility of cell-specific gene and cell surface protein expression differences when incorporated into a ML framework for detecting PSA, with most of the ML algorithms considered in this study classifying PSA, PSO, and healthy subjects or distinguishing just between PSA and PSO subjects with >70% average accuracy on either gene or protein features. Combining both types of features reduced overall model performance, possibly due to differences in the magnitude of interindividual or technical variation in the detected expression of these feature types, which may not be accurately accounted for by the subject-averaged expression data we used for model training and testing. Nevertheless, our study expands the number of potential biomarkers and cell types relevant to diagnosing PSA and understanding its biology.

We note that our data, being derived solely from peripheral blood immune populations, cannot address whether these cell types are also present in the synovium, whether they may instead represent systemic responses to cutaneous inflammation in PSA subjects, or the extent that they arise from either a migration of cells between blood and tissue compartments or an overall expansion or reduction in specific cell subsets. Also, since PSA patients in our study already have established arthritic disease, our data may not capture early or ephemeral biomarkers of disease that may appear in PSO patients who eventually develop PSA. Future investigations combining single cell multiomics on blood, skin, and joint immune populations with a longitudinal follow-up of PSO patients [as employed by Abji et al. (47)] may help overcome these limitations.

## Data Availability Statement

The datasets presented in this study can be found in online repositories. The names of the repository/repositories and accession number(s) can be found below: NCBI GEO, accession no: GSE194315 (https://www.ncbi.nlm.nih.gov/geo/query/acc.cgi?acc=GSE194315).

## Ethics Statement

The studies involving human participants were reviewed and approved by University of California San Francisco IRB. The patients/participants provided their written informed consent to participate in this study.

## Author Contributions

WL and LG conceived and supervised the project. JH, DP, MCas, MCal, H-WC, MCh, SY, EB, MH, TB, MM, LG, and WL recruited study subjects and performed clinical annotation. H-WC and Z-MH performed experimental procedures. JL, SK, DC, and WL performed data analysis. CY provided technical and analytic support. JL, SK, and WL wrote and revised the manuscript. All authors contributed to the article and approved the submitted version.

## Funding

This study was funded by grants from the National Psoriasis Foundation to WL. This study was also supported by PREMIER, a NIH/NIAMS P30 Center for the Advancement of Precision Medicine in Rheumatology at UCSF (P30AR070155​). WL is supported by NIH grant R01AR078688. JL is supported by NIH grant T32AR007175. CY is supported by NIH grants R01AR071522, R01AI136972, U01HG012192, and the Chan Zuckerberg Initiative, and is an investigator at the Chan Zuckerberg Biohub and is a member of the Parker Institute for Cancer Immunotherapy (PICI). The funders above were not involved in the study design, collection, analysis, interpretation of data, the writing of this article or the decision to submit it for publication.

## Conflict of Interest

Author WL has received funding from Abbvie, Amgen, Janssen, Leo, Novartis, Pfizer, Regeneron, and TRex Bio. Author CY is a Scientific Advisory Board member for and holds equity in Related Sciences and ImmunAI, a consultant for and holds equity in Maze Therapeutics, and a consultant for TReX Bio. Author CY has received funding from Chan Zuckerberg Initiative, Chan Zuckerberg Biohub, and Genentech. Author LG has received funding from Novartis, Pfizer, UCB, AbbVie, Eli Lilly, Gilead, Janssen, Novartis, Pfizer and UCB.

The remaining authors declare that the research was conducted in the absence of any commercial or financial relationships that could be construed as a potential conflict of interest.

## Publisher’s Note

All claims expressed in this article are solely those of the authors and do not necessarily represent those of their affiliated organizations, or those of the publisher, the editors and the reviewers. Any product that may be evaluated in this article, or claim that may be made by its manufacturer, is not guaranteed or endorsed by the publisher.

## References

[1] Mease PJ Gladman DD Papp KA Khraishi MM Thaçi D Behrens F. Prevalence of Rheumatologist-Diagnosed Psoriatic Arthritis in Patients With Psoriasis in European/North American Dermatology Clinics. J Am Acad Dermatol (2013) 69:729–35. doi: 10.1016/j.jaad.2013.07.023 23981683

[2] Haroon M Gallagher P FitzGerald O. Diagnostic Delay of More Than 6 Months Contributes to Poor Radiographic and Functional Outcome in Psoriatic Arthritis. Ann Rheumatic Dis (2015) 74:1045–50. doi: 10.1136/annrheumdis-2013-204858 24525911

[3] Pennington SR FitzGerald O. Early Origins of Psoriatic Arthritis: Clinical, Genetic and Molecular Biomarkers of Progression From Psoriasis to Psoriatic Arthritis. Front Med (2021) 8:723944. doi: 10.3389/fmed.2021.723944 PMC8416317 34485351

[4] Stuart PE Nair RP Tsoi LC Tejasvi T Das S Kang HM. Genome-Wide Association Analysis of Psoriatic Arthritis and Cutaneous Psoriasis Reveals Differences in Their Genetic Architecture. Am J Hum Genet (2015) 97:816–36. doi: 10.1016/j.ajhg.2015.10.019 PMC4678416 26626624

[5] FitzGerald O Haroon M Giles JT Winchester R. Concepts of Pathogenesis in Psoriatic Arthritis: Genotype Determines Clinical Phenotype. Arthritis Res Ther (2015) 17:115. doi: 10.1186/s13075-015-0640-3 25948071 PMC4422545

[6] O’Brien-Gore C Gray EH Durham LE Taams LS Kirkham BW. Drivers of Inflammation in Psoriatic Arthritis: The Old and the New. Curr Rheumatol Rep (2021) 23:40. doi: 10.1007/s11926-021-01005-x 33909160

[7] Cretu D Gao L Liang K Soosaipillai A Diamandis EP Chandran V. Differentiating Psoriatic Arthritis From Psoriasis Without Psoriatic Arthritis Using Novel Serum Biomarkers. Arthritis Care Res (2018) 70:454–61. doi: 10.1002/acr.23298 28586166

[8] Chandran V Cook RJ Edwin J Shen H Pellett FJ Shanmugarajah S. Soluble Biomarkers Differentiate Patients With Psoriatic Arthritis From Those With Psoriasis Without Arthritis. Rheumatology (2010) 49:1399–405. doi: 10.1093/rheumatology/keq105 20421218

[9] Leijten E Tao W Pouw J van Kempen T Olde Nordkamp M Balak D. Broad Proteomic Screen Reveals Shared Serum Proteomic Signature in Patients With Psoriatic Arthritis and Psoriasis Without Arthritis. Rheumatology (2021) 60:751–61. doi: 10.1093/rheumatology/keaa405 PMC7850582 32793974

[10] Purcell S Neale B Todd-Brown K Thomas L Ferreira MAR Bender D. PLINK: A Tool Set for Whole-Genome Association and Population-Based Linkage Analyses. Am J Hum Genet (2007) 81:559–75. doi: 10.1086/519795 PMC1950838 17701901

[11] Chang CC Chow CC Tellier LC Vattikuti S Purcell SM Lee JJ. Second-Generation PLINK: Rising to the Challenge of Larger and Richer Datasets. GigaScience (2015) 4:7. doi: 10.1186/s13742-015-0047-8 25722852 PMC4342193

[12] Danecek P Auton A Abecasis G Albers CA Banks E DePristo MA. The Variant Call Format and VCFtools. Bioinformatics (2011) 27:2156–8. doi: 10.1093/bioinformatics/btr330 PMC3137218 21653522

[13] Kang HM Subramaniam M Targ S Nguyen M Maliskova L McCarthy E. Multiplexed Droplet Single-Cell RNA-Sequencing Using Natural Genetic Variation. Nat Biotechnol (2018) 36:89–94. doi: 10.1038/nbt.4042 29227470 PMC5784859

[14] Hao Y Hao S Andersen-Nissen E Mauck WM Zheng S Butler A. Integrated Analysis of Multimodal Single-Cell Data. Cell (2021) 184:3573–3587.e29. doi: 10.1016/j.cell.2021.04.048 34062119 PMC8238499

[15] DePasquale EAK Schnell DJ Van Camp P-J Valiente-Alandí Í Blaxall BC Grimes HL. DoubletDecon: Deconvoluting Doublets From Single-Cell RNA-Sequencing Data. Cell Rep (2019) 29:1718–27.e8. doi: 10.1016/j.celrep.2019.09.082 31693907 PMC6983270

[16] Hoque N Singh M Bhattacharyya DK. EFS-MI: An Ensemble Feature Selection Method for Classification. Complex Intelligent Syst (2018) 4:105–18. doi: 10.1007/s40747-017-0060-x

[17] Yoo Y. Hyperparameter Optimization of Deep Neural Network Using Univariate Dynamic Encoding Algorithm for Searches. Knowledge-Based Syst (2019) 178:74–83. doi: 10.1016/j.knosys.2019.04.019

[18] Patrick MT Stuart PE Raja K Gudjonsson JE Tejasvi T Yang J. Genetic Signature to Provide Robust Risk Assessment of Psoriatic Arthritis Development in Psoriasis Patients. Nat Commun (2018) 9:4178. doi: 10.1038/s41467-018-06672-6 30301895 PMC6177414

[19] Andrés Cerezo L Šumová B Prajzlerová K Veigl D Damgaard D Nielsen CH. Calgizzarin (S100A11): A Novel Inflammatory Mediator Associated With Disease Activity of Rheumatoid Arthritis. Arthritis Res Ther (2017) 19:79. doi: 10.1186/s13075-017-1288-y 28446208 PMC5405489

[20] Abraham SM Clark AR. Dual-Specificity Phosphatase 1: A Critical Regulator of Innate Immune Responses. Biochem Soc Trans (2006) 34:1018–23. doi: 10.1042/BST0341018 17073741

[21] Szremska AP Kenner L Weisz E Ott RG Passegué E Artwohl M. JunB Inhibits Proliferation and Transformation in B-Lymphoid Cells. Blood (2003) 102:4159–65. doi: 10.1182/blood-2003-03-0915 12907453

[22] Ellebedy AH Jackson KJL Kissick HT Nakaya HI Davis CW Roskin KM. Defining Antigen-Specific Plasmablast and Memory B Cell Subsets in Human Blood After Viral Infection or Vaccination. Nat Immunol (2016) 17:1226–34. doi: 10.1038/ni.3533 PMC5054979 27525369

[23] Koncz G Hueber A-O. The Fas/CD95 Receptor Regulates the Death of Autoreactive B Cells and the Selection of Antigen-Specific B Cells. Front Immunol (2012) 3:207. doi: 10.3389/fimmu.2012.00207 22848207 PMC3404404

[24] Tu M Cai L Zheng W Su Z Chen Y Qi S. CD164 Regulates Proliferation and Apoptosis by Targeting PTEN in Human Glioma. Mol Med Rep (2017) 15:1713–21. doi: 10.3892/mmr.2017.6204 PMC5364976 28259931

[25] Gil M Pak H-K Lee A-N Park S-J Lee Y Roh J. CD99 Regulates CXCL12-Induced Chemotaxis of Human Plasma Cells. Immunol Lett (2015) 168:329–36. doi: 10.1016/j.imlet.2015.10.015 26522646

[26] Lu D Liu L Ji X Gao Y Chen X Liu Y. The Phosphatase DUSP2 Controls the Activity of the Transcription Activator STAT3 and Regulates TH17 Differentiation. Nat Immunol (2015) 16:1263–73. doi: 10.1038/ni.3278 26479789

[27] Capasso M Durrant LG Stacey M Gordon S Ramage J Spendlove I. Costimulation *via* CD55 on Human CD4+ T Cells Mediated by CD97. J Immunol (Baltimore Md : 1950) (2006) 177:1070–7. doi: 10.4049/jimmunol.177.2.1070 16818763

[28] Miller ML Mashayekhi M Chen L Zhou P Liu X Michelotti M. Basal NF- B Controls IL-7 Responsiveness of Quiescent Naive T Cells. Proc Natl Acad Sci (2014) 111:7397–402. doi: 10.1073/pnas.1315398111 PMC4034246 24799710

[29] Castellino F Huang AY Altan-Bonnet G Stoll S Scheinecker C Germain RN. Chemokines Enhance Immunity by Guiding Naive CD8+ T Cells to Sites of CD4+ T Cell–Dendritic Cell Interaction. Nature (2006) 440:890–5. doi: 10.1038/nature04651 16612374

[30] Pedrosa E Carretero-Iglesia L Boada A Colobran R Faner R Pujol-Autonell I. CCL4L Polymorphisms and CCL4/CCL4L Serum Levels Are Associated With Psoriasis Severity. J Invest Dermatol (2011) 131:1830–7. doi: 10.1038/jid.2011.127 21614014

[31] Peng Y-M van de Garde MDB Cheng K-F Baars PA Remmerswaal EBM van Lier RAW. Specific Expression of GPR56 by Human Cytotoxic Lymphocytes. J leukocyte Biol (2011) 90:735–40. doi: 10.1189/jlb.0211092 21724806

[32] Watanabe T Masuyama J Sohma Y Inazawa H Horie K Kojima K. CD52 is a Novel Costimulatory Molecule for Induction of CD4+ Regulatory T Cells. Clin Immunol (2006) 120:247–59. doi: 10.1016/j.clim.2006.05.006 16797237

[33] Dhuban KB Bartolucci S D’Hennezel E Piccirillo CA. Signaling Through Gp130 Compromises Suppressive Function in Human FOXP3+Regulatory T Cells. Front Immunol (2019) 10:1532. doi: 10.3389/fimmu.2019.01532 31379810 PMC6657659

[34] Ueyama A Imura C Fusamae Y Tsujii K Furue Y Aoki M. Potential Role of IL-17-Producing CD4/CD8 Double Negative αβ T Cells in Psoriatic Skin Inflammation in a TPA-Induced STAT3C Transgenic Mouse Model. J Dermatol Sci (2017) 85:27–35. doi: 10.1016/j.jdermsci.2016.10.007 27810232

[35] Sherlock JP Joyce-Shaikh B Turner SP Chao C-C Sathe M Grein J. IL-23 Induces Spondyloarthropathy by Acting on ROR-γt+ CD3+CD4-CD8- Entheseal Resident T Cells. Nat Med (2012) 18:1069–76. doi: 10.1038/nm.2817 22772566

[36] King KY Goodell MA. Inflammatory Modulation of HSCs: Viewing the HSC as a Foundation for the Immune Response. Nat Rev Immunol (2011) 11:685–92. doi: 10.1038/nri3062 PMC4154310 21904387

[37] Leijten EF van Kempen TS Olde Nordkamp MA Pouw JN Kleinrensink NJ Vincken NL. Tissue-Resident Memory CD8+ T Cells From Skin Differentiate Psoriatic Arthritis From Psoriasis. Arthritis Rheumatol (2021) 73:1220–32. doi: 10.1002/art.41652 PMC8362143 33452865

[38] Wang J Zhang S-X Hao Y-F Qiu M-T Luo J Li Y-Y. The Numbers of Peripheral Regulatory T Cells are Reduced in Patients With Psoriatic Arthritis and Are Restored by Low-Dose Interleukin-2. Ther Adv Chronic Dis (2020) 11:204062232091601. doi: 10.1177/2040622320916014 PMC7236566 32523664

[39] Pan X Yuan X Zheng Y Wang W Shan J Lin F. Increased CD45RA+FoxP3low Regulatory T Cells With Impaired Suppressive Function in Patients With Systemic Lupus Erythematosus. PloS One (2012) 7:e34662. doi: 10.1371/journal.pone.0034662 22506043 PMC3323568

[40] Diani M Casciano F Marongiu L Longhi M Altomare A Pigatto PD. Increased Frequency of Activated CD8+ T Cell Effectors in Patients With Psoriatic Arthritis. Sci Rep (2019) 9:1–10. doi: 10.1038/s41598-019-47310-5 31350460 PMC6659700

[41] Penkava F Velasco-Herrera MDC Young MD Yager N Nwosu LN Pratt AG. Single-Cell Sequencing Reveals Clonal Expansions of Pro-Inflammatory Synovial CD8 T Cells Expressing Tissue-Homing Receptors in Psoriatic Arthritis. Nat Commun (2020) 11:4767. doi: 10.1038/s41467-020-18513-6 32958743 PMC7505844

[42] Sgambelluri F Diani M Altomare A Frigerio E Drago L Granucci F. A Role for CCR5(+)CD4 T Cells in Cutaneous Psoriasis and for CD103(+) CCR4(+) CD8 Teff Cells in the Associated Systemic Inflammation. J Autoimmun (2016) 70:80–90. doi: 10.1016/j.jaut.2016.03.019 27068801

[43] Casciano F Diani M Altomare A Granucci F Secchiero P Banfi G. CCR4+ Skin-Tropic Phenotype as a Feature of Central Memory CD8+ T Cells in Healthy Subjects and Psoriasis Patients. Front Immunol (2020) 11:529. doi: 10.3389/fimmu.2020.00529 32318062 PMC7147166

[44] Chiu YG Shao T Feng C Mensah KA Thullen M Schwarz EM. CD16 (Fcrγiii) as a Potential Marker of Osteoclast Precursors in Psoriatic Arthritis. Arthritis Res Ther (2010) 12:R14. doi: 10.1186/ar2915 20102624 PMC2875642

[45] Suzuki E Maverakis E Sarin R Bouchareychas L Kuchroo VK Nestle FO. Adamopoulos IE. T Cell-Independent Mechanisms Associated With Neutrophil Extracellular Trap Formation and Selective Autophagy in IL-17a-Mediated Epidermal Hyperplasia. J Immunol (Baltimore Md : 1950) (2016) 197:4403–12. doi: 10.4049/jimmunol.1600383 PMC5123839 27798153

[46] Adamopoulos IE Suzuki E Chao C-C Gorman D Adda S Maverakis E. IL-17A Gene Transfer Induces Bone Loss and Epidermal Hyperplasia Associated With Psoriatic Arthritis. Ann Rheumatic Dis (2015) 74:1284–92. doi: 10.1136/annrheumdis-2013-204782 PMC4229480 24567524

[47] Abji F Pollock RA Liang K Chandran V Gladman DD. Brief Report: CXCL10 Is a Possible Biomarker for the Development of Psoriatic Arthritis Among Patients With Psoriasis. Arthritis Rheumatol (2016) 68:2911–6. doi: 10.1002/art.39800 27389865

