# Supplementary material for: Combined Single Cell Transcriptome and Surface Epitope Profiling Identifies Potential Biomarkers of Psoriatic Arthritis and Facilitates Diagnosis via Machine Learning
Source: Front Immunol. 2022 Mar 2;13:835760. doi: 10.3389/fimmu.2022.835760 (PMC8924042; doi:10.3389/fimmu.2022.835760)
Supplement: Supplementary file 1 [file DataSheet_1.pdf]

Supplementary Figure 1

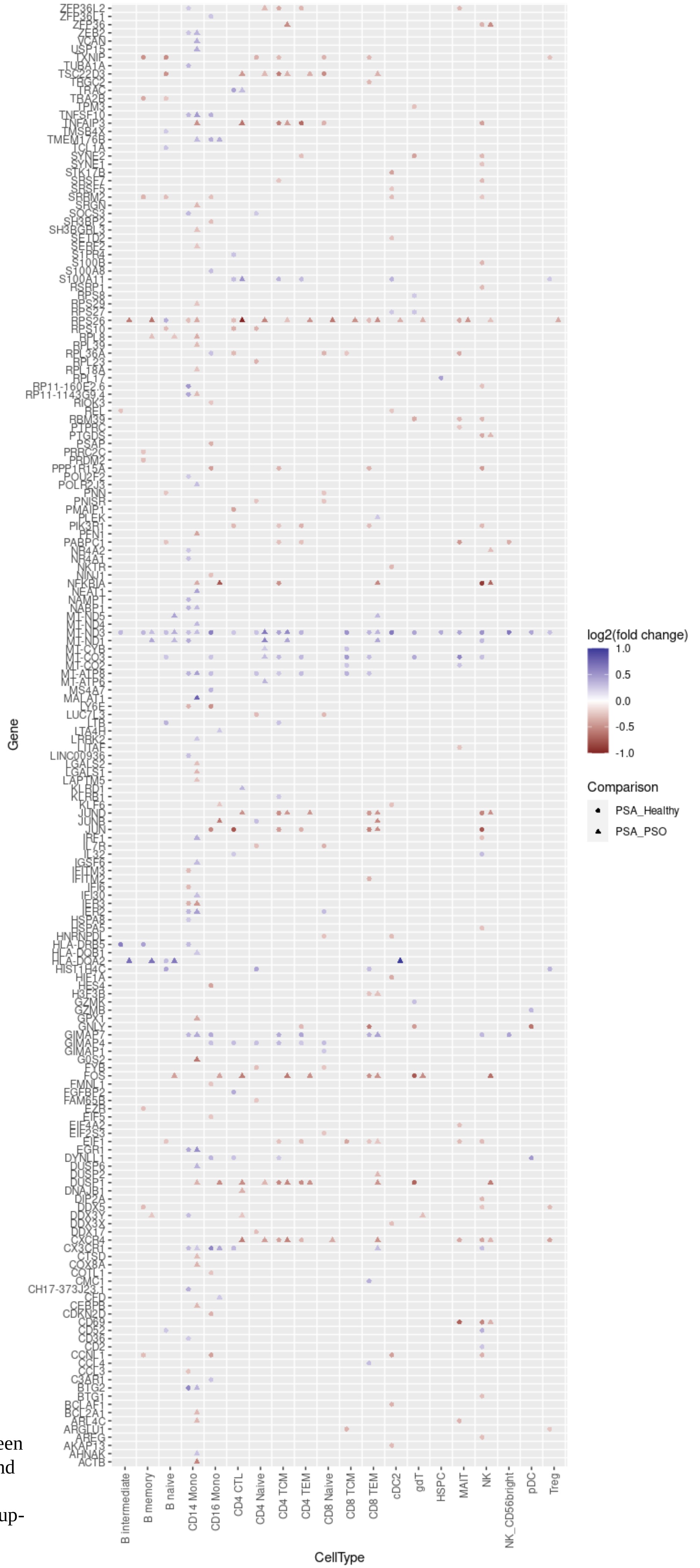

Supplementary Figure 1. Differentially expressed genes among cell subsets. DEGs between PSA and PSO (circles) or PSA and healthy (triangles) are shown for each cell subset. Color indicates up- or down-regulation.

Supplementary Figure 2

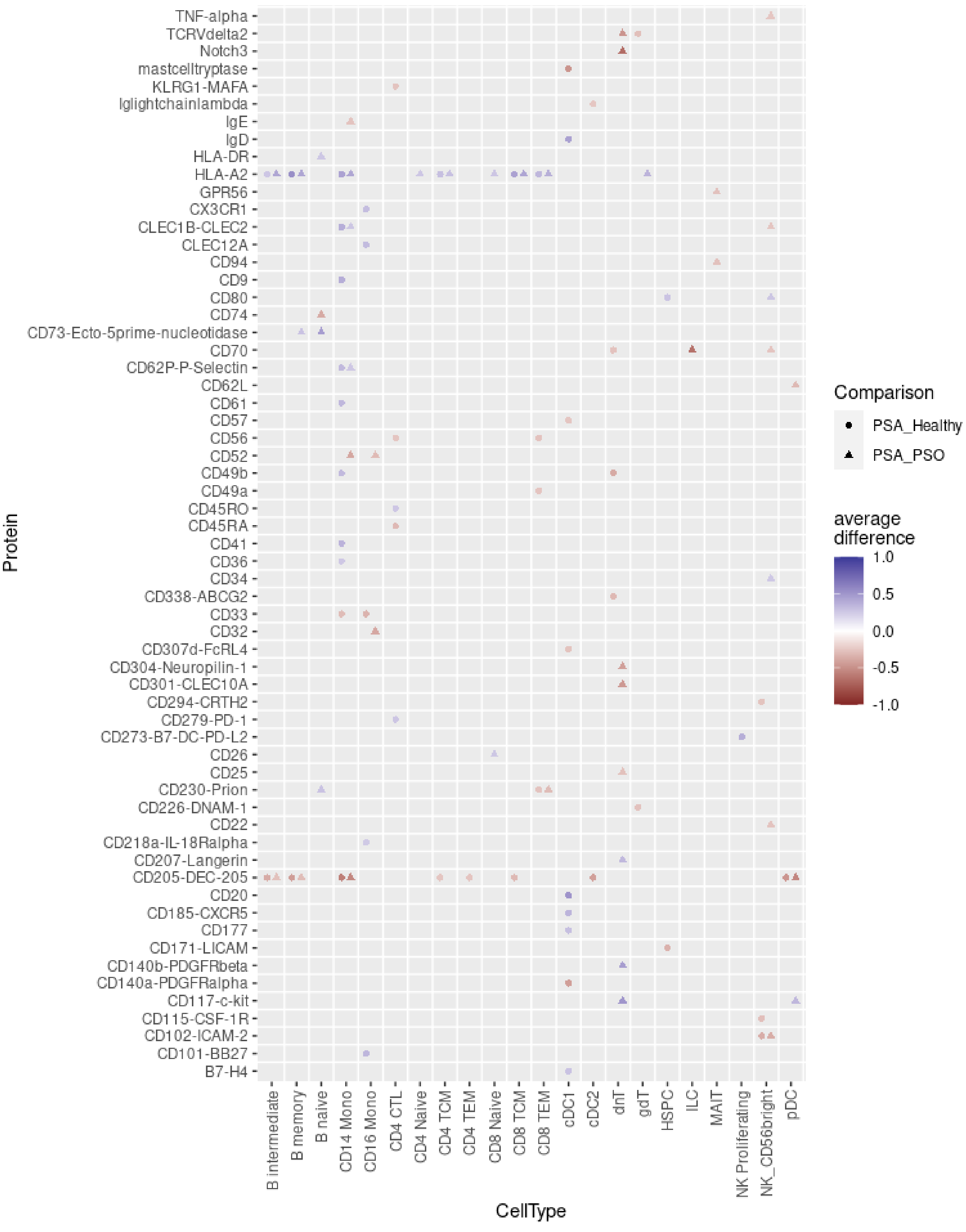

Supplementary Figure 2. Differentially expressed cell surface proteins among cell subsets. DEPs between PSA and PSO (circles) or PSA and healthy (triangles) are shown for each cell subset. Color indicates up- or down-regulation.

Supplementary Figure 3

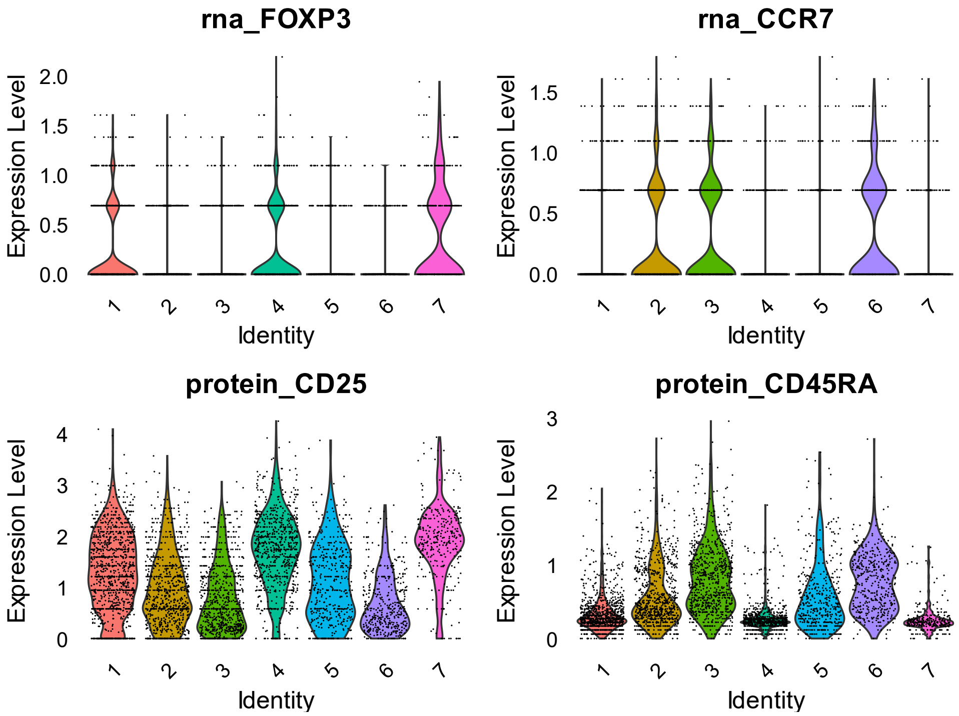

Supplementary Figure 3. Comparison of T cell gene and protein markers across Treg clusters.

Supplementary Figure 4

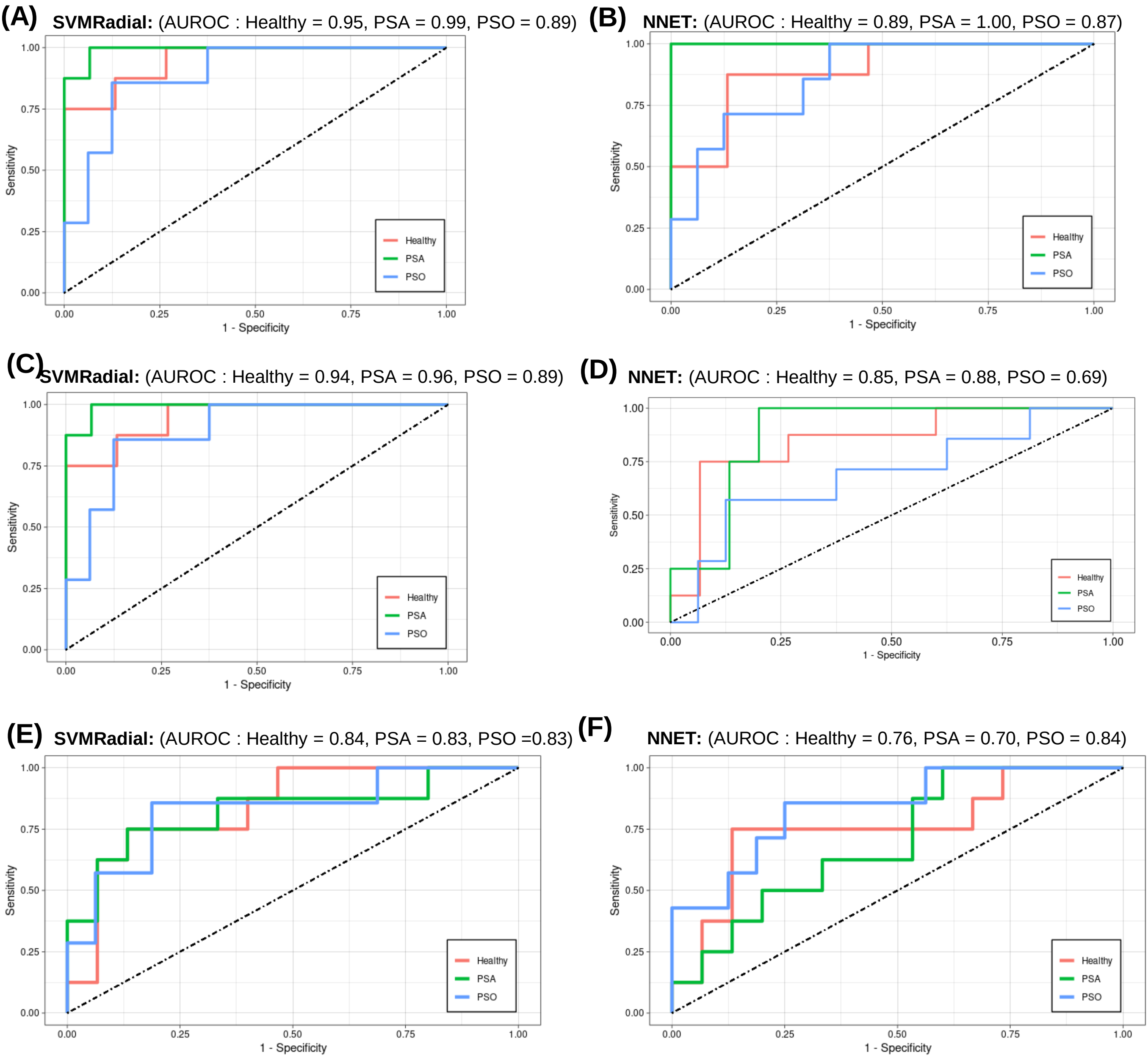

Supplementary Figure 4. Independent external validation using test set of healthy vs. PSA vs. PSO patient state classification based on scRNA, ADT and combined (scRNA + ADT). ROC curves for SVMRadial and NNET classifiers based on (A,B) DEGs, (C,D) DEPs, or (E,F) both.

Supplementary Figure 5

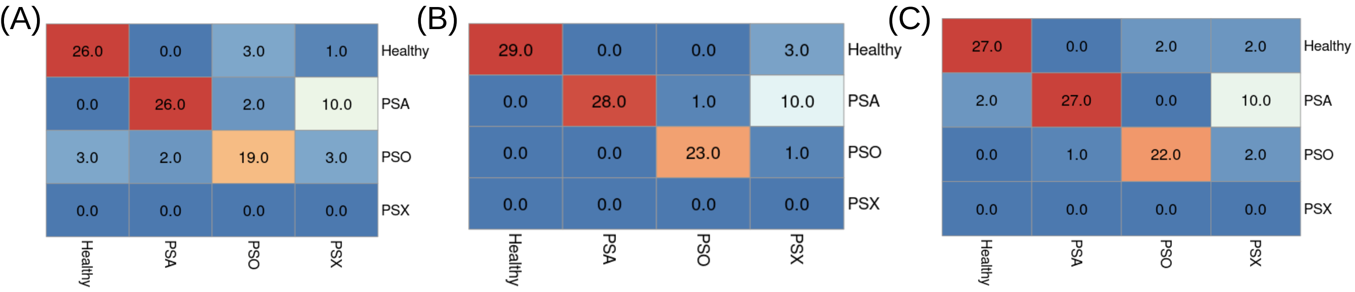

Supplementary Figure 5. External cross-validation of RF model using PSX samples. Confusion matrices showing the number of subjects from each group (in rows) assigned to a predicted group (in columns) by RF classifiers based on (A) DEGs (B) DEPs, or (C) both.

Supplementary Figure 6

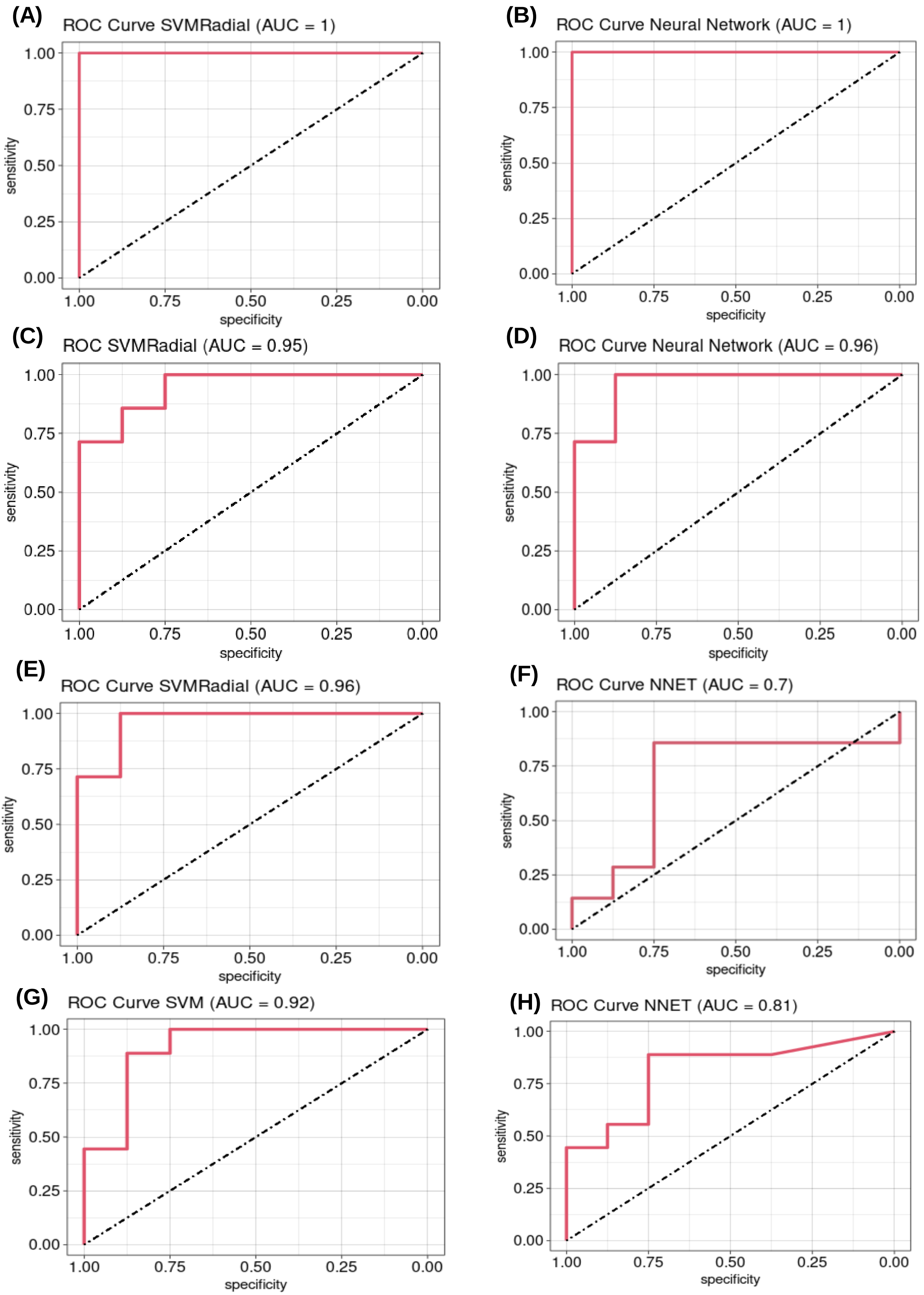

Supplementary Figure 6. PSA and PSO patient classification based on scRNA, ADT, and combined (scRNA + ADT) expression, along with PSA-associated SNPs. AUROC curves for SVMRadial and NNET classifiers based on (A,B) DEGs, (C,D) DEPs, (E,F) both, and (G,H) 200 PSA-associated SNPs.
